# Violations of newly-learned predictions elicit two distinct P3 components

**DOI:** 10.3389/fnhum.2014.00374

**Published:** 2014-06-10

**Authors:** Abigail Noyce, Robert Sekuler

**Affiliations:** ^1^Department of Psychology, Brandeis UniversityWaltham, MA, USA; ^2^Department of Psychology and Brain Sciences, Boston UniversityBoston, MA, USA; ^3^Volen Center for Complex Systems, Brandeis UniversityWaltham, MA, USA

**Keywords:** expectation, P3, EEG, sequence learning, working memory

## Abstract

Sensitivity to the environment's sequential regularities makes it possible to predict upcoming sensory events. To investigate the mechanisms that monitor such predictions, we recorded scalp EEG as subjects learned to reproduce sequences of motions. Each sequence was seen and reproduced four successive times, with occasional deviant directions of motion inserted into otherwise-familiar and predictable sequences. To dissociate the neural activity associated with encoding new items from that associated with detecting sequence deviants, we measured ERPs to new, familiar, and deviant sequence items. Both new and deviant sequence items evoked enhanced P3 responses, with the ERP to deviant items encompassing both P300-like and Novelty P3-like subcomponents with distinct timing and topographies. These results confirm that the neural response to deviant items differs from that to new items, and that unpredicted events in newly-learned sequences are identified by processes similar to those monitoring stable sequential regularities.

## Introduction

The human brain frequently operates in feedforward mode, exploiting previously-experienced regularities to build expectations for future events. This proactive operation facilitates perceptual processing (Bar, 2009) and allows appropriate behaviors to be prepared and executed in a timely fashion (e.g., Kowler, 1989; Maryott et al., 2011). Among the richest regularities available to the brain are ones entailed not in single isolated events, but in event sequences. In fact, the brain constructs and continuously updates its representation of such sequential regularities in an obligatory and effortless manner (Johnson and Donchin, 1982; Kimura et al., 2011; Sternberg and McClelland, 2012).

In order to benefit fully from the advantages of feedforward operation, the brain must have a mechanism to detect events that violate its expectations, and to trigger appropriate responses to those violations (Winkler, 2007). Such responses might include heightening attention to the unexpected event, modifying or delaying a prepared behavior, or updating the brain's representation of the regularity at hand. Successful prediction monitoring must also distinguish prediction errors that are due to stochasticity or noise from errors that reflect a genuine change in the rules governing the environment (Yu and Dayan, 2005; Nassar et al., 2010).

Event-related brain potentials (ERPs) provide a direct measure of neural activity time-locked to specific events (Luck, 2005). Their temporal precision makes ERPs a useful tool for studying the neural reaction to events within a sequential structure. Among ERP components, the P3, a positive-going deflection seen at central electrodes from 300 to 500 ms after a novel or surprising stimulus, has often been used to study the neural response to unexpected events (Squires et al., 1976; Linden, 2005; Polich, 2007). Many authors have interpreted the P3 to reflect processes related to updating the contents of working memory, such as contextual updating, event categorization, stimulus evaluation, or changing a course of action (Goldstein et al., 2002; van Zuijen et al., 2006; Ridderinkhof et al., 2009). The P3 is thought to be generated in large part by the anterior cingulate cortex, a region whose dense connections to sensory, limbic, and prefrontal areas make it ideally situated to perform prediction monitoring (Ridderinkhof et al., 2004; Linden, 2005; Crottaz-Herbette and Menon, 2006). Some authors have identified separable subcomponents within this late positivity, and have proposed that the long list of P3-eliciting situations may be divided according to which subcomponents are elicited. In particular, the degree to which a event is unusual generally enhances positivity at scalp locations that are more anterior, while the degree to which an event requires a response or is otherwise task-relevant tends to enhance positivity at scalp locations that are more centro-parietal (Goldstein et al., 2002; Gaeta et al., 2003; Rüsseler et al., 2003; Nieuwenhuis et al., 2005; Barcelo et al., 2006; Polich, 2007; Ferdinand et al., 2008).

While the P3 has historically been studied in oddball paradigms, where a stream of stimuli contains both frequent and infrequent stimulus exemplars (e.g., Courchesne et al., 1975; Squires et al., 1975; van Zuijen et al., 2006), it has also been studied in sequence-learning tasks. Stimuli that deviate from an established sequential structure elicit a larger P3 response than stimuli that conform to the sequential structure (Rüsseler and Rösler, 2000; Schlaghecken et al., 2000; Rüsseler et al., 2003; Ferdinand et al., 2008). The studies demonstrating this P3 enhancement have required subjects to learn only a single sequence per block of many trials, allowing subjects to encode and maintain one sequential regularity that remained valid for an extended period of time. However, humans engage in predictive cognition on many time-scales, from very fast motor control adjustments to years-long planning.

Here, we investigated the neural mechanisms underlying sequence learning and prediction monitoring in a setting where the governing regularities were short-lived. To do so, we adopted the task used by Maryott et al. (2011) to study behavioral responses to deviant events embedded in complex sequential structures that frequently changed. In that study, subjects had to remember and reproduce short sequences of movements, each approximately 5 s long. Each sequence was seen and reproduced several times in succession, but occasionally a deviant, unexpected item was inserted into a well-learned sequence. Their participants successfully incorporated the deviant items into their representation of the sequence, and even showed a slight benefit in reproducing those deviant items. Note that Maryott et al.'s task differs from traditional sequence-learning paradigms in that it required that the brain frequently update its representation of the governing sequential structure, as a new sequence began every 60–90 s.

To investigate whether this dynamic, changing context would alter the brain's response to a deviant sequence item, we recorded EEG signals from subjects as they performed a variant of the task used by Maryott et al., and measured ERPs to new, familiar, and deviant sequence items. Here, we show that both new and deviant sequence items evoke a larger P3 than familiar sequence items do, but that the topography and time course of the P3 enhancement elicited by new items differs from that elicited by deviant items. This difference reflects the distinction between the task-relevance P300/P3b and the Novelty P3/P3a ERP subcomponents (Goldstein et al., 2002; Linden, 2005; Polich, 2007), with new items enhancing only a P300-like component and deviant items enhancing both P300-like and Novelty P3-like components. Our results show that (1) the neural response to deviant sequence elements in a frequently-updated environment is broadly similar to that seen in more stable settings, and (2) the neural processes that identify and respond to prediction-violating events differ from those that merely encode a new stimulus.

## Methods

All experimental procedures were approved by the Brandeis Committee for Protection of Human Subjects.

### Subjects

Twelve young adults (7 female, ages 19–28) participated in this experiment. All were naïve to the task; all were right-handed. Informed consent was obtained from each subject.

### Experimental task

To induce and measure sequence learning, we asked participants to observe and reproduce pseudo-random motion trajectories. Each trajectory was presented four successive times, and each participant saw 128 different trajectories.

**Figure 1:**
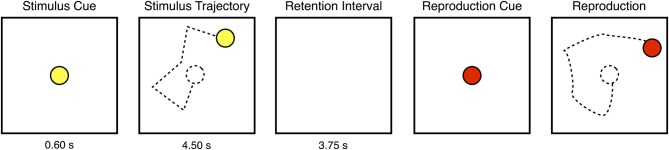
**One presentation of a motion sequence stimulus**. At the start of each presentation, a yellow disk appeared at the center of the display before beginning to move in a series of five, connected linear segments without leaving any visible trail. After enacting the five movements, the disk disappeared from view. After a retention interval, a second disk appeared, signaling the subject to begin reproducing from memory the path that had been previously traveled by the yellow disk. Each trial consisted of four such presentations.

On each presentation of a trajectory, a yellow disk traversed a path comprising five connected linear motion segments. Figure 1 illustrates the sequence of events within one such presentation. Each segment of the trajectory was 1 cm (approximately 1° visual angle) in length, and the disk moved at a constant speed of 2 cm per second, taking 0.50 s to travel the length of each segment. After each segment, the disk paused for 0.40 s before resuming its motion in a changed direction. The yellow disk then disappeared from view. After a retention interval of 3.75 s, a second disk appeared, cueing the subject to move a handheld stylus over the surface of a graphics tablet (31 × 24 cm; Intuos 3, Wacom, Vancouver, WA) in order to reproduce from memory the sequence of disk motions that had just been seen. During the reproduction, the disk's motion was yoked to the movement of the stylus' tip on the graphics tablet. No other feedback was provided. Note that neither the stimulus nor the reproduction disk left a visible trail while moving across the computer display. Subjects viewed the stimuli from a distance of approximately 57 cm, and were instructed to maintain fixation on a central cross, and to refrain from blinking, while the stimulus disk was on the screen.

Each trial's quasi-random sequence of five motion segments was generated by the algorithm described by Agam et al. (2005). The direction of a sequence's initial motion was chosen randomly, and the direction change at each “corner” of the trajectory was between 30° and 150°. These changes in direction could be clockwise or counter-clockwise, with equal probability. The motions comprising any sequence were constrained by several additional rules: Motion segments were not permitted to intersect, could not come within one-half a segment's length of intersecting, and could not extend beyond the boundaries of the display area.

### Design and procedure

**Figure 2:**
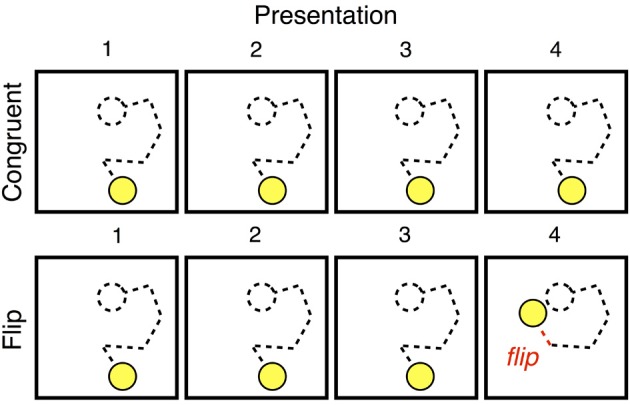
**The sequence of presentations that made up a ***Congruent*** trial or ***Flip*** trial**. In our analyses, we considered the first presentation of a stimulus to be *new*, the fourth presentation of a **Congruent** trial to be *familiar*, and the “flipped” segment at the end of a **Flip** trial to be *deviant*.

On every trial, that trial's unique trajectory was presented four times, with participants reproducing the sequence after each such presentation. Each set of four presentations constituted either a **Congruent** trial, in which all four presentations of a sequence were identical to one another, or a **Flip** trial, in which one motion segment changed direction on the sequence's fourth (and final) presentation. Figure 2 illustrates these two conditions. On the fourth and final presentation of a **Flip** trial, the final segment of the established trajectory was replaced by a segment whose direction of motion was exactly 180° opposed.

We operationalized three types of events: *new, familiar*, and *deviant*. *New* events were motion segments on their first presentation; *familiar* events were segments on their fourth presentation, and *deviant* events were the “flipped” final segment of the final presentation on **Flip** trials. Note that new and deviant events are still congruent with the task context, and thus quite similar to other events within the experiment.

Subject completed four 45-min experimental sessions, in which they observed and reproduced 32 different trajectories four times each. In each session, the first two trials were **Congruent** trials, followed by 20 **Congruent** and 10 **Flip** trials, block-randomized so that **Flip** trials were more evenly distributed. Forty trials (approximately 31%) were **Flip** trials; the remaining 88 were **Congruent**. This approximates the ratio of Flip to Congruent trials used by Maryott et al. (2011), in which subjects did not anticipate the flips (as demonstrated by subjects' anticipatory eye movements). Subjects were never informed that some trials would be **Flip** trials.

### Behavioral data analysis

A two-step algorithm quantified the fidelity of each reproduction (Agam et al., 2005; Maryott et al., 2011). It first used pauses and direction changes to divide the reproduction into segments, and then estimated the direction of each such segment by fitting a line to its beginning and end points. Reproduction accuracy was quantified by directional error: the absolute angular difference between the direction of a motion segment in the reproduction and the direction of the corresponding segment in the stimulus exemplar.

Note that this segmentation algorithm's output is invalid if it divides a reproduced trajectory into a number of segments that differs from the number in the exemplar trajectory. To increase the likelihood that reproductions would be successfully divided into five motion segments, we instructed subjects to try to produce the same number of segments that had been in the stimulus (five) and to, insofar as possible, draw straight lines with corners between them. These instructions allowed the segmentation algorithm to successfully divide over 90% of trials.

### Electrophysiological recording

A high-density EEG system (Electrical Geodesics, Inc., Eugene, OR) with 129 electrodes sampled scalp electroencephalographic signals at 250 Hz using a high-impedance amplifier. Signals were recorded for later, off-line analysis. All channels were adjusted for scalp impedance below 50 kΩ; after 12 trials, channels were again adjusted for impedance below 50 kΩ scalp impedance.

### EEG analysis

EEG data were cleaned and analyzed in the EEGLAB (Delorme and Makeig, 2004) and FieldTrip (Oostenveld et al., 2011) toolboxes for Matlab (The Mathworks, Inc., Natick, MA). Data were re-referenced to the average voltage, bandpass filtered to between 0.25 and 75 Hz, and divided into epochs for each segment of each presentation. Every such epoch extended from 200 ms before that segment's disk motion onset to 600 ms after. Data were visually inspected for muscle artifacts, eye movements, and bad channels; epochs containing such artifacts were rejected. Independent components analysis was used to isolate eye blink activity, which was subtracted from the data. Finally, data were again visually inspected for artifacts not corrected by the previous two processes. After cleaning, data were averaged across trials and sessions to create a subject average ERP for combinations of **condition** (**Congruent** or **Flip**), **segment** (**one, two, three, four**, or **five**), and **presentation** (**one, two, three**, or **four**). ERPs were timelocked to the onset of motion at the beginning of each segment.

For each of the following investigations, we used a data-driven, non-parametric clustering approach (Maris and Oostenveld, 2007) to select time windows and electrodes for analysis. The FieldTrip toolbox includes software implementing this approach. It first calculates Student's *t* for each electrode and time point, and identifies clusters of time- and/or space-adjacent electrodes with |*t*| > *t*_crit_. Criterion *t*-values were selected by the experimenters after considering several factors, including the degrees of freedom of the comparison, the magnitude of the difference between the conditions, and the degree of spatial and temporal specificity desired. In order to maintain spatial and temporal specificity when the differences between conditions were large, we used a more-conservative value of *t*_crit_.

For each cluster, the *t*-scores of its member electrodes and time-points were summed, giving a cluster score that reflected both the extent of the cluster (in space and time) and the magnitude of the difference between the conditions. A reference distribution of test statistics was generated by randomly permuting the data across the two conditions being compared, computing such scores for each resulting cluster, and taking the largest such cluster score on each of 1000 permutations. Where cluster-wise *p*-values are reported, they have been derived by comparing the empirically-obtained cluster score to such a reference distribution.

Grand average ERPs for each comparison of interest were created by averaging across subjects and across the electrodes identified as part of the cluster. After identifying time windows at which the two conditions differed, but before investigating response topography, we corrected for amplitude differences by dividing each electrode's voltage by the room mean squared electrode voltage within that condition (McCarthy and Wood, 1985; Picton et al., 2000).

To measure the differences between neural responses to deviant sequence items and those to familiar sequence items, we computed ERPs to **segment five** of **presentation four** on **Congruent** and **Flip** trials. Note that this is the segment and presentation on which the “flip” occurs on **Flip** trials. After cleaning and preprocessing, the ERPs to the familiar segment included a mean of 64.33 epochs per subject (*SD* = 10.48, minimum = 41), and the ERPs to the deviant segment included a mean of 32.08 epochs per subject (*SD* = 4.23, minimum = 22). We will denote these segments as **familiar** and **deviant**. When viewing the deviant sequence items, subjects needed to first identify that their prediction had been disconfirmed and then encode the segment's direction of movement. The neural response to deviant segments should thus reflect both increased encoding demands and the prediction-monitoring processes that trigger such new encoding.

To dissociate encoding a new item from detecting unexpected events, we directly compared the neural responses to new and deviant sequence items. We computed ERPs to **segment five** of **Flip** trials on both **presentation one** and **presentation four**. After cleaning and preprocessing, the ERPs to the new segment included a mean of 32.08 epochs per subject (*SD* = 4.56, minimum = 22). We will refer to these two segments as **new** and **deviant**. On **presentation one** of these trials, when viewing new items, subjects could not predict any segment's direction of motion, while on **presentation four**, subjects had acquired predictions about the disk's motion, which were then violated by the deviant stimulus.

## Results

### Behavior

**Figure 3:**
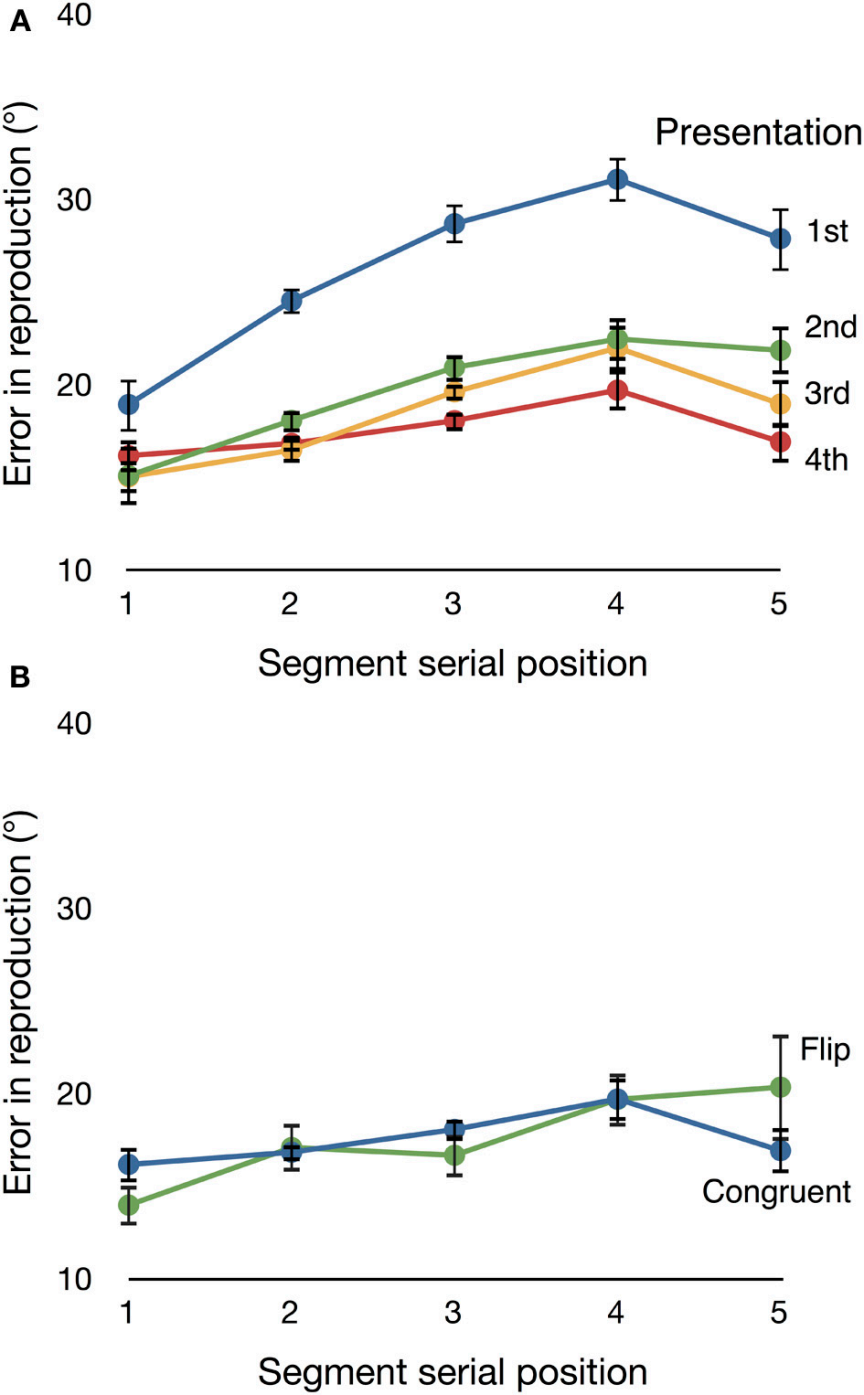
**(A)** Mean directional error on **Congruent** trials across the five segments in a motion sequence. Data are displayed separately for each of the four presentations of a sequence. Subjects' accuracy improved over repeated presentations. **(B)** Mean directional error on **Flip** and **Congruent** trials for the fourth (final) presentation. The two conditions do not differ significantly. Error bars are repeated-measures standard error (Morey, 2008).

Figure 3A shows subjects' reproduction accuracy on **Congruent** trials. We ran a 5 × 4 ANOVA with factors **segment** (**one, two, three, four**, or **five**) and **presentation** (**one, two, three**, or **four**), and a Greenhouse–Geisser correction for sphericity. The ANOVA showed a main effect of **segment** [*F*_(4, 44)_ = 8.955, ϵ = 0.409, *p* = 0.003, partial *η*^2^ = 0.449], and a **segment** by **presentation** interaction [*F*_(12, 132)_ = 6.310, ϵ = 0.054, *p* =0.048, partial *η*^2^ = 0.364]. There was also a main effect of **presentation** [*F*_(3, 33)_ = 38.866, ϵ = 0.580, *p* < 0.001, partial *η*^2^ = 0.779]. Follow-up analyses showed that the improvement in reproduction accuracy from **presentation one** to **presentation two** was significant [*F*_(1, 11)_ = 62.654, *p* < 0.001, partial *η*^2^ = 0.850], but the change from **presentation two** to **three**, and that from **three** to **four**, were not. The largest learning effects occur after only one presentation.

We next confirmed that differences in the neural responses to familiar and deviant items did not reflect significant differences in the accuracy with which subjects encoded **Flip** and **Congruent** trials. Figure 3B shows mean directional error for each trial type on **presentation four** (that is, the presentation on which these two trial types differed). We found no significant main effect of condition [**Flip** vs. **Congruent**, *F*_(1, 11)_ = 2.554, *p* = 0.138, partial *η*^2^ = 0.189]. These results replicate the finding of Maryott et al. (2011) and confirm that subjects can successfully incorporate unexpected events into their planning and execution of a reproduced motion sequence.

A fully detailed analysis of the behavioral results is beyond the scope of this paper, as such analyses of closely-related experiments have been previously published (Agam et al., 2007; Maryott et al., 2011), and the focus of this paper is on the neural responses to new, familiar, and deviant sequence items.

### ERPs

**Figure 4:**
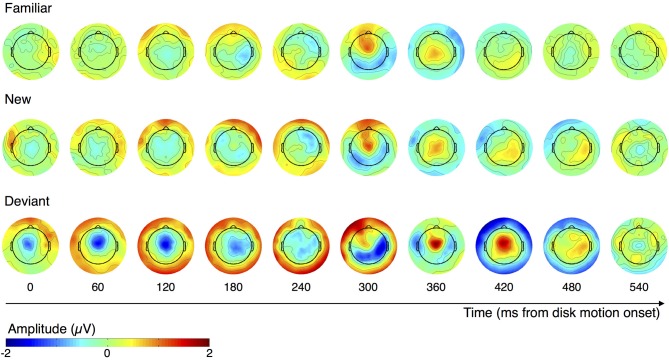
**Topographical plots of ERP amplitude across the scalp, over the course of familiar, new, and deviant motion segments**. Each plot shows the mean distribution of voltage during the 60 ms window centered on its labeled time point.

Figure 4 illustrates the changes in spatial distribution of ERP amplitude following disk motion onset as subjects observe familiar (top), new (middle), and deviant (bottom) sequence items. Each topographical plot displays the mean voltage during a 60 ms window. These plots suggest that the neural responses to new and familiar items are similar, with only the amplitude differing, while the neural response to deviant items is substantially different. We turned to two directed comparisons to quantify this observation.

#### Familiar and deviant items

**Figure 5:**
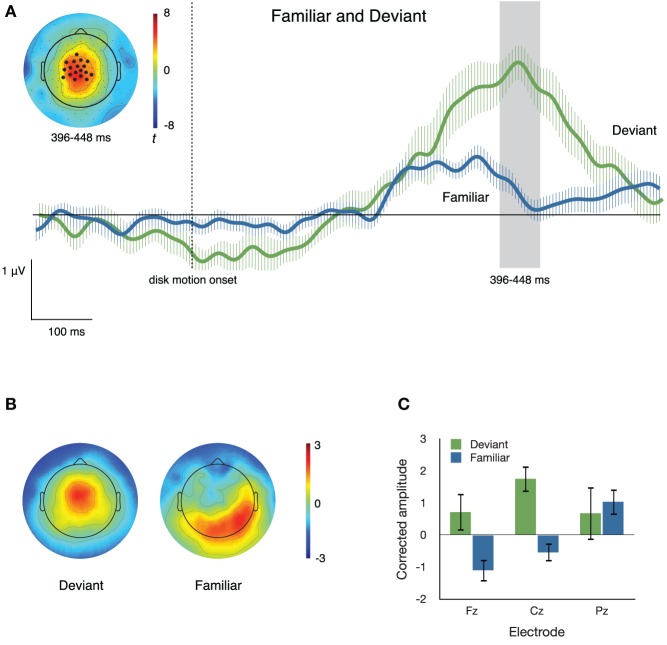
**(A)** ERPs to familiar and deviant sequence items at a cluster of central electrodes. The inset topographical plot shows the locations of the 18 electrodes making up the significant cluster, and the distribution across the scalp of *t* values at 396–448 ms after disk motion onset. The traces show ERPs at the cluster, timelocked to disk motion onset of each segment. Error bars are repeated-measures standard error of the mean, and do not reflect the output of the statistical significance testing process. **(B)** Topographical plots of the spatial distribution of voltage from 396 to 448 ms after disk motion onset, after correcting for overall amplitude differences between conditions. Topographies appear to differ substantially. **(C)** Mean corrected amplitude from 396 to 448 ms after disk motion onset, at three midline electrodes. The response to deviant items is significantly more positive-going than the response to familiar items.

Figure 5A illustrates the neural activity that accompanies familiar and deviant sequence items. We used the data-driven approach described above to identify the cluster of electrodes that best captured (*p*< 0.001) the difference between the segment that “flips” on **Flip** trials and its counterpart on **Congruent** trials. The cluster was derived using *t*_crit_ = 3.106, the critical *t*-value at α = 0.01, *df* = 11. The resulting cluster consisted of 18 electrodes that were more positive-going in response to deviant than to familiar segments, from 396 to 448 ms after disk motion onset. The inset in Figure 5A depicts the distribution of *t*-scores between ERPs to deviant and to familiar segments during that time window and the locations of the electrodes comprising this cluster; the traces in the main body of Figure 5A depict ERPs at the cluster, elicited by deviant and familiar motion segments.

To confirm differences in topography between the neural responses to familiar and deviant items, we normalized by the root mean squared electrode voltage in order to correct for amplitude differences between conditions. Figure 5B illustrates the distribution of voltage across the scalp after such correction. The corrected amplitude at three midline electrodes over the time window from 396 to 448 ms after disk motion onset is shown in Figure 5C. We ran a 2 × 3 ANOVA with factors **condition** (**deviant** and **familiar**) and **electrode** (**Fz, Cz**, and **Pz**) on the corrected amplitudes. There was a main effect of condition [*F*_(1, 11)_ = 8.249, *p* = 0.015, partial *η*^2^ = 0.429], no main effect of electrode [*F*_(2, 22)_ = 2.162, *p* = 0.139, partial *η*^2^ = 0.164], and no condition by electrode interaction [*F*_(2, 22)_ = 1.976, *p* = 0.162, partial *η*^2^ = 0.152]. These results confirm what Figure 5C suggests: amplitude at these three midline electrodes is higher in response to deviant than to familiar segments, even after correcting for overall amplitude of the response. Thus, the activity of the response to deviant segments is concentrated at these central regions, consistent with what's shown in both the uncorrected topographical plots in Figure 4 and the corrected topographical plots in Figure 5B.

#### New and deviant items

**Figure 6:**
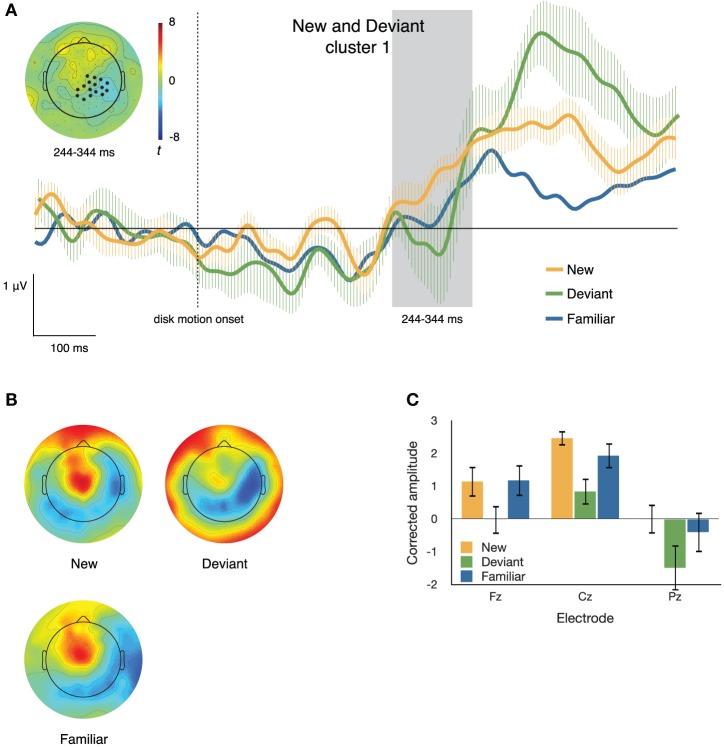
**(A)** ERPs to new, deviant, and familiar sequence items at one of the two clusters differentiating these conditions. The inset topographical plot shows the locations of the electrodes making up the slightly right-lateralized centro-parietal cluster and the distribution of *t* values at 244–344 ms after disk motion onset; the traces show ERPs at those electrodes. **(B)** Topographical plots of the spatial distribution of voltage from 244 to 344 ms after disk motion onset, after correcting for overall amplitude differences between conditions. Deviant items appear to elicit markedly different topographies from new items. **(C)** Mean corrected amplitude from 344 to 444 ms after disk motion onset, at three midline electrodes. The response to new items is significantly more positive-going than the response to deviant items.

**Figure 7:**
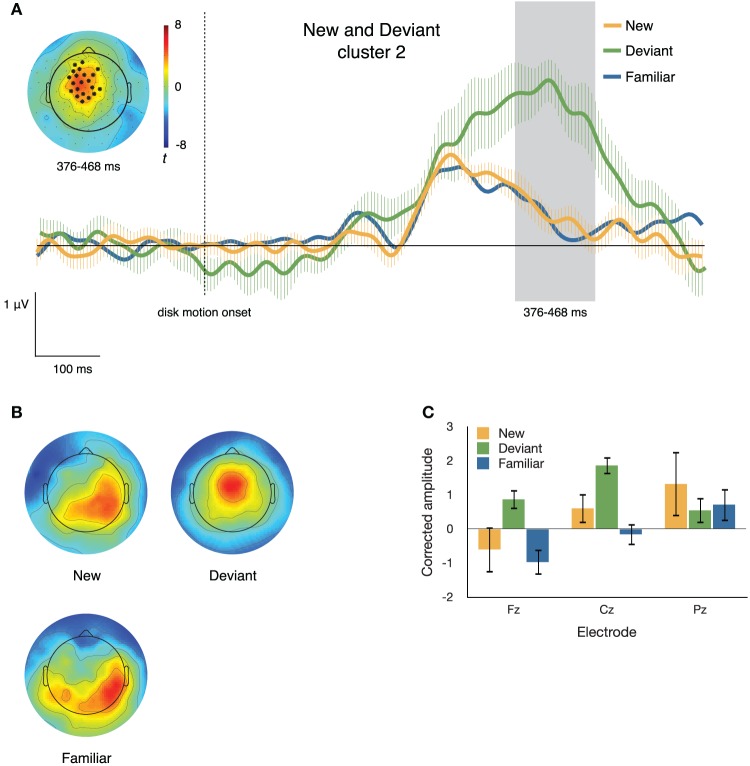
**(A)** ERPs to new, deviant, and familiar sequence items at the second of the two clusters differentiating these conditions. The inset topographical plot shows the locations of the electrodes making up the slightly right-lateralized centro-parietal cluster and the distribution of *t* values at 376–468 ms after disk motion onset; the traces show ERPs at those electrodes. **(B)** Topographical plots of the spatial distribution of voltage from 376 to 468 ms after disk motion onset, after correcting for overall amplitude differences between conditions. Deviant items appear to elicit markedly different topographies from new items. **(C)** Mean corrected amplitude from 376 to 468 ms after disk motion onset, at three midline electrodes. There is a significant interaction between electrode and condition.

Figures 6, 7 illustrate the differences between neural responses to deviant and to new sequence items. The clustering algorithm described above identified two electrode clusters and time windows that differed between ERPs to a new item and ERPs to a deviant item in the same sequential position. These clusters were derived using *t*_crit_ = 2.201, the critical *t*-value at α = 0.05, *df* = 11.

***Cluster 1***. Figure 6 depicts the first (by time) resulting cluster (*p* = 0.025), which consisted of 16 electrodes that were more negative-going on deviant than on new segments from 244 to 344 ms after disk motion onset. The inset of Figure 6A shows the distribution of *t*-scores between ERPs to the two segments during that time window (negative values imply the neural response to deviant items is more negative) and the locations of the electrodes comprising this cluster; the traces in the main body of Figure 6A depict ERPs at that cluster, time locked to disk motion onset. For comparison, the figure also shows traces of the ERP to the equivalent familiar segment.

To confirm differences in topography between the neural responses to familiar and deviant items during this time window, we corrected for the amplitude differences between conditions. Figure 6B illustrates the distribution of voltage across the scalp after such correction. The corrected amplitude at three midline electrodes over the time window from 244 to 344 ms after disk motion onset is shown in Figure 6C. We ran a 2 × 3 ANOVA with factors **condition** (**new** and **deviant**) and **electrode** (**Fz, Cz**, and **Pz**). There was a main effect of condition [*F*_(1, 11)_ = 48.272, *p* < 0.001, partial *η*^2^ = 0.814] and a main effect of electrode [*F*_(2, 22)_ = 4.594, *p* = 0.022, partial *η*^2^ = 0.295], but no significant condition by electrode interaction [*F*_(2, 22)_ = 0.168, *p* = 0.847, partial *η*^2^ = 0.015]. Figure 6C shows that at all three electrodes, the response elicited by new items is more positive than that response to deviant items at this time window, and that pattern does not vary across electrodes.

***Cluster 2***. The second cluster (*p* = 0.002) consisted of 26 electrodes that were more positive-going on deviant than on new segments, from 376 to 468 ms after disk motion onset for the segment. The inset of Figure 7A depicts the distribution of *t*-scores between the two conditions during this time window and the locations of the electrodes comprising this cluster. The traces in the main body of Figure 7A show ERPs at the cluster, time locked to disk motion onset.

To confirm differences in topography between the neural responses to new and deviant items during this second time window, we corrected for the amplitude differences between conditions. Figure 7B illustrates the distribution of voltage across the scalp after such correction. The corrected amplitude at three midline electrodes over the time window from 376 to 468 ms after disk motion onset is shown in Figure 7C. We ran a 2 × 3 ANOVA with factors **condition** (**new** and **deviant**) and **electrode** (**Fz, Cz**, and **Pz**). There was a marginal main effect of condition [*F*_(1, 11)_ = 3.776, *p* = 0.078, partial *η*^2^ = 0.256], no main effect of electrode [*F*_(2, 22)_ = 2.339, *p* = 0.120, partial *η*^2^ = 0.175], and a significant condition by electrode interaction [*F*_(2, 22)_ = 7.129, *p* = 0.004, partial *η*^2^ = 0.393]. The amplitude at each electrode over this time window is shown in Figure 7B. At Fz and Cz, the response to deviant items is more positive-going than the response to new items; at Pz the effect is reversed. *Post hoc t*-tests confirmed this interaction, *t*_(11)_ = 3.700, *p* = 0.003.

These results suggest that the two electrode clusters depicted in Figures 6A, 7A likely capture effects corresponding to two different ERP sub-components. The centro-parietal cluster illustrated in Figure 6 occurs due to a parietal negativity elicited by deviant items relative to new items. At the time window from 244 to 344 ms after disk motion onset, it appears that the neural response to new items has begun to ramp up toward a broad positive peak at these electrodes The neural response to deviant items, on the other hand, is still negative-going at 244–344 ms, but its later positivity, at this cluster, is larger than that elicited by new items. Note that the late positivity at these electrodes has highest amplitude in response to deviant sequence items, next highest in response to new sequence items, and is lowest in response to familiar items.

The pattern shown in Figure 7 is very different. The second cluster is found at the time window encompassing a fronto-central peak in the neural response to deviant items. This peak is much higher than the positivity peak seen at this cluster of electrodes in the neural responses to new and familiar items. It's particularly important to note that the ERP traces associated with new and familiar items at this cluster do not differ from each other, while the traces associated with the deviant items are substantially more positive-going.

## Discussion

We recorded high-density scalp EEG while subjects performed a visuomotor sequence-learning task, with occasional deviant elements inserted into recently-learned sequences. Subjects successfully reproduced the sequences, including the deviant items, demonstrating an ability to incorporate unexpected events into their planning and execution of a motion sequence. Because the sequence changed every 60–90 s, subjects had to dynamically update their representation of the relevant sequential structure. In order to characterize the neural mechanisms that respond to unpredictability within these newly-learned sequences, we measured ERPs to new, familiar, and deviant sequence items. Relative to both new and familiar items, deviant items elicited strong P3 enhancement over fronto-central areas that began about 300 ms after item onset and peaked sharply around 400–450 ms. Deviant items also elicited a broad P3 enhancement over centro-parietal areas that began around 400 ms after disk motion onset and peaked around 450 ms. New items elicited similar P3 enhancement at the centro-parietal cluster relative to familiar items, although that response was smaller and earlier than the response to deviant items. It's important to note that there is an unmistakeable P3-like central positivity elicited by familiar items as well; this positivity is, however, smaller than that elicited by new or deviant items.

Note that we created ERPs by time-locking to the onset of disk motion at the beginning of each segment. While there is a substantial literature on motion-onset evoked potentials (see review by Kuba et al., 2007), including reports of motion-onset P3 activity (Kuba et al., 1998; Agam and Sekuler, 2007), it is likely that the neural response to motion onset is more variable in both magnitude and latency than the response to a luminance onset or offset, reducing the signal to noise ratio of our data (Luck, 2005). It is therefore possible that some early or transient ERP components are not adequately captured. Nonetheless, we believe that our P3 results are, if anything, strengthened by their robustness in this situation.

### Neural responses to sequence deviants

This experiment fills an important gap in the previous work on the neural response to sequence deviants, most of which has been done using a serial reaction time task (SRTT). In the SRTT, subjects make speeded key presses to a stream of letters or other stimuli (Nissen and Bullemer, 1987). When a repeating sequence of letters is embedded within the stream, subjects respond more quickly to those repeating items, and some subjects develop explicit knowledge of the sequence's presence and structure. Subjects who have such explicit knowledge show an enhanced P3 to letters that violate the established sequence, but subjects without such knowledge do not (Schlaghecken et al., 2000; Ferdinand et al., 2008). Similarly, subjects who are instructed that an underlying sequence exists show an enhanced P3 (Rüsseler et al., 2003).

Interestingly, in speeded sequential learning tasks, deviant events interfere with behavioral performance (Nattkemper and Prinz, 1997; Rüsseler and Rösler, 2000; Schlaghecken et al., 2000). On the other hand, we saw no or minimal behavioral effects of the deviant events in our task. One possible explanation for this is that the neural representation of dynamic motion trajectories differs substantially from the neural representation of sequences of individual key presses or static items, as in the SRTT. However, we believe that the difference reflects the nature of the response required. Rather than make speeded single responses, our subjects must encode the entire sequence into short-term memory, maintain that representation for several seconds, and then generate a complete reproduction. The reaction-time effects of deviant sequence items that have been seen in the SRTT are thus unlikely to occur in such a task setting.

Rüsseler and Rösler (2000) drew on Nattkemper and Prinz (1997)'s SRTT variant in which multiple stimuli mapped onto each response key. This allowed them to separate the neural response to perceptual deviants (new letter but same response) from the neural response to items that were deviant in both the perceptual and motor domains (new letter and different keypress). They found an enhanced P3 only to these latter double deviants, and concluded that the P3 effect reflects the need to change or update a response rather than merely detecting an unexpected stimulus (see also Goldstein et al., 2002). In our task, participants are explicitly aware of the sequential structure, as they must memorize and reproduce it. Thus, a deviant sequence item in our study requires participants to change a planned motor output and to encode information about the new event. Our finding that the P3 is enhanced by deviant sequence items is consistent with Rüsseler and Rösler's hypothesis. A recent related study measured ERPs while subjects observed short sequences of pictures depicting the steps of everyday actions, ending in either a correct execution of the action or an error (de Bruijn et al., 2007). Observed errors elicited an enhanced P3 relative to correct executions. In de Bruijn et al.'s task, subjects were presumably drawing on their previous knowledge about the sequential structure making up the actions; observed errors deviate from this structure but do not require a response from the participant.

### P3 findings and interpretation

Previous work on the P3 has identified two distinct subcomponents with very similar timing (e.g., Squires et al., 1975; Spencer et al., 2001; Goldstein et al., 2002; Linden, 2005; Polich, 2007). The slightly earlier subcomponent, the Novelty P3 (sometimes called the P3a), is centered over fronto-central regions, and is elicited by deviant stimuli that are salient and low-probability, regardless of whether they require a response (Goldstein et al., 2002). The second subcomponent, the P300, is more posterior, centered over centro-parietal electrodes. The P300 is elicited by task-relevant stimuli such as targets. Deviant sequence elements in the SRTT enhance the P300 (also sometimes called P3b) subcomponent (Schlaghecken et al., 2000; Rüsseler et al., 2003; Ferdinand et al., 2008). This seemingly-clear theoretical distinction has been made more complex by later work demonstrating Novelty P3-like activity evoked by task-relevant cues and targets (e.g., Gaeta et al., 2003; Barcelo et al., 2006). Polich (2007) has proposed a model of the cognitive processes corresponding with the Novelty P3/P3a and the P300/P3b subcomponents in which the Novelty P3 indexes processes involved in allocating or switching attention to an unlikely event, and the P300 reflects changing the contents of working memory. Our findings are consistent with this model.

Our results support the presence of two distinct late positivities enhanced by deviant sequence items relative to new and familiar items. One, a right-lateralized centro-parietal ERP had the highest amplitude in response to deviant items, the next highest in response to new items, and the lowest in response to familiar items (Figure 6). This response appears to be similar to the P300 (or P3b), in that it is generally posterior of Cz and comprises a broad positive peak. If it is a variant of the P3b, that suggests that it reflects the process of encoding a motion segment into working memory in preparation for reproducing it (Polich, 2007). Over repeated presentations of the same sequence, the working-memory adjustments required after each succeeding presentation are reduced, leading to the decreased amplitude for familiar items relative to the other two conditions (as seen by Agam et al., 2010), even though the segment is still task-relevant. We find the right-lateralization of this positivity particularly intriguing, given the right-hemisphere bias in visual attention (Corbetta and Shulman, 2002).

The second late positivity enhanced by deviant sequence items is a more fronto-central ERP with sustained high amplitude associated with deviant sequence items and a much smaller positivity associated with new and familiar items (Figures 5, 7). This response appears to be similar to the Novelty P3 (or P3a) in its sharper peak and true fronto-central distribution. These characteristics, and its enhancement to deviant items while not dissociating new from familiar items, strongly suggest that this positivity enhancement is specifically associated with expectation violation, and may reflect either the shift of attention to the unexpected event, or a process of inhibiting or rejecting a representation and motor plan that were actively maintained in working memory (Yu and Dayan, 2005; Polich, 2007; Vossel et al., 2009).

The identification of an enhanced Novelty P3/P3a-like subcomponent in the response to deviant sequence items is especially interesting given that the deviant events in our study are quite different from the usual eliciting conditions for a Novelty P3 (e.g., Goldstein et al., 2002; Polich and Comerchero, 2003; Polich, 2007). Our deviant items are motion segments, at the same speed as other segments, within the same general task constraints; they are unexpected only in that their direction of motion is 180° different from that which subjects are presumed to expect. Observing a component that bears a strong resemblance to the Novelty P3 in this setting is thus a surprising and important finding, and may expand our understanding of novelty and deviance processing in tasks that require more elaborate cognitive processing.

### Conclusions

Differences between the neural responses to new and to deviant stimuli highlight the importance of prediction-monitoring in cognition. On the surface, new and deviant items are quite similar: both require the subject to perceive and encode a direction of motion that has not been previously observed, in order to reproduce it from memory. The difference between the two types of events lies in their contexts. When viewing a deviant sequence item, participants have strong predictions about the disk's direction of motion; when viewing a new item, their predictions are much more uniformly distributed. The differences in neural response between these two conditions therefore reflects the effects of these predictions on perceiving and encoding the element.

Although our results illuminate the relationship between sequential structure, unpredictability, and the P3, our study does have two important limitations. First, because participants were explicitly aware of both the sequential structure of the task and the points at which new trials (and thus new sequences) began, these findings cannot be extended to explain the mechanisms by which people identify sequential structure in a continuous, unbroken stream of sensory input, nor to explain the processes by which people identify changes in that structure. Second, because deviant items in this paradigm always differed from the familiar items by 180°, we cannot say anything about the effect of the magnitude of prediction violations. Both of these questions will need to be investigated before the relationship between expected unpredictability, unexpected unpredictability, and the subcomponents of the P300 can be fully described.

In summary, we have shown that, when learning motion sequences, people show distinct neural responses to new stimuli, familiar stimuli, and stimuli that deviate from the governing sequence. The neural responses to a deviant sequence item differ from those to a new sequence item, further supporting the hypothesis that identifying prediction errors is a cognitive process. Finally, our results extend previous work on monitoring sequential regularities and show that the neural mechanisms involved are similar when the sequential structure is frequently updated and when it is stable over time.

## Funding

Supported by CELEST, an NSF Science of Learning Center (SBE-0354378), and by NIH Training Grant T32GM084907.

### Conflict of interest statement

The authors declare that the research was conducted in the absence of any commercial or financial relationships that could be construed as a potential conflict of interest.
